# Vascular anatomy of kiwi fruit and its implications for the origin of carpels

**DOI:** 10.3389/fpls.2013.00391

**Published:** 2013-10-16

**Authors:** Xue-Min Guo, Xiao Xiao, Gui-Xi Wang, Rong-Fu Gao

**Affiliations:** ^1^College of Life Science and Technology, Hebei Normal University of Science and TechnologyQinhuangdao, China; ^2^Research Institute of Forestry, Chinese Academy of Forestry/Key Laboratory of Tree Breeding and Cultivation, State Forestry AdministrationBeijing, China; ^3^College of Life Science and Technology, Beijing Forestry UniversityBeijing, China

**Keywords:** angiosperm, placenta, origin, comparative anatomy, kiwi fruit

## Abstract

Kiwi fruit is of great agricultural, botanical, and economic interest. The flower of kiwi fruit has axile placentation, which is typical for Actinidiaceae. Axile placentation is thought derived through fusion of conduplicate carpels with marginal placentation according to the traditional doctrine. Recent progress in angiosperm systematics has refuted this traditional doctrine and placed ANITA clade rather than Magnoliaceae as the basalmost clade. However, the former traditional doctrine stays in the classrooms as the only teachable theory for the origin of carpels. To test the validity of this doctrine, we performed anatomical study on kiwi fruit. Our study indicates that the placenta has a vascular system independent of that of the ovary wall, the ovules/seeds are attached to the placenta that is a continuation of floral axis enclosed by the lateral appendages that constitute the ovary wall, and there are some amphicribral bundles in the center of placenta and numerous amphicribral bundles supplying ovules/seeds in kiwi fruit. The amphicribral vascular bundles supplying the ovules/seeds are comparable to those usually seen in branches, but not comparable to those seen in leaves or their derivatives. This comparison indicates that the placenta in kiwi fruit cannot be derived from the fusion of collateral ventral bundles of conduplicate carpels, as suggested by traditional doctrine. Instead the vascular organization in placenta of kiwi suggests that the placenta is a shoot apex-bearing ovules/seeds laterally. This conclusion is in line with the recently raised Unifying Theory, in which the placenta is taken as an ovule-bearing branch independent of the ovary wall (carpel in strict sense). Similar vascular organization in placenta has been seen in numerous isolated taxa besides kiwi fruit. Therefore whether such a pattern is applicable for other angiosperms is an interesting question awaiting answering.

## INTRODUCTION

Vascular bundles are the main transport channels of water and nutrients in the fruit, and play a very important role in fruit development and quality formation (Choat et al., 2009; Zhang et al., 2009; Measham et al., 2010) in addition to their systematic significance. The growth of fleshy fruits involves a balance between the supply or withdrawal of water via the vascular tissue, and losses to transpiration (Morandi et al., 2007; Clearwater et al., 2011). Kiwi fruit is of great agricultural, botanical, and economic interest. In kiwi fruit, as in most fruits, storage quality is related to calcium concentration and many disorders are associated with low fruit calcium concentration, and calcium transport to the fruit is exclusively via the xylem – calcium is not phloem mobile (Marschner, 1983; White, 2001; Dichio et al., 2003). Kiwi fruit vasculature is similar to that of the ovary at anthesis (Schmid, 1978). Many of the fruit vascular bundles are considerably larger than those of the flower owing to the formation of secondary vascular tissue, particularly secondary xylem (Habart, 1974; Schmid, 1978). However, these studies on kiwi fruit vascular bundles are not systematic and the evolutionary implications of the vascular bundles are rarely touched. Here the distributions, shapes, types, and structure of vascular bundles in kiwi fruit were systematically observed in order to provide a basis for studies on material transport and accumulation, mechanism of quality formation. Also the implications of vascular anatomy for the forming of carpel are explored.

## MATERIALS AND METHODS

Female flowers and some 150-day-old fruits of *Actinidia deliciosa *var. *deliciosa* ‘Qinmei’ were collected from an orchard in Fangshan, Beijing in 2012, and two 150-day-old fruits were sectioned in both the transverse and longitudinal planes and then photographed. Samples were taken from central placenta (CP), peripheral placenta (PP), and outer pericarp (OP) at the base, in the middle and near the top of other fruits. The samples were fixed with FAA and then used in the preparation of 8-μm thick paraffin-sections following the routine method. These sections were critically examined tracing the vascular bundles and photographed using a Nikon-E400 microscope with a Nikon E-995 digital camera.

## RESULTS

**FIGURE 1:**
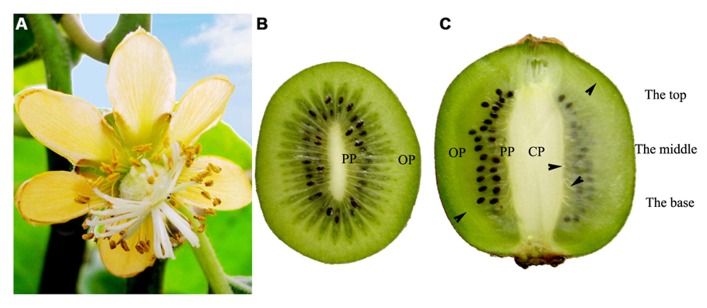
**Female flower and fruits of *Actinidia deliciosa *var *deliciosa *‘Qinmei.’ (A)** A female flower, showing syncarpous pistil constituted by many carpels. **(B)** Mid-cross section of fruit, showing many carpels around the axile placenta. **(C)** Longitudinal mid-section of fruit. Note the major vascular bundles (arrowheads) of fruit. CP, central placenta; PP, peripheral placenta; OP, outer pericarp.

Kiwi fruit is derived from compound pistil ovary, and its vascular bundles are mainly distributed in three distinct regions: CP, PP, and OP (**Figures 1A–C**).

**FIGURE 2:**
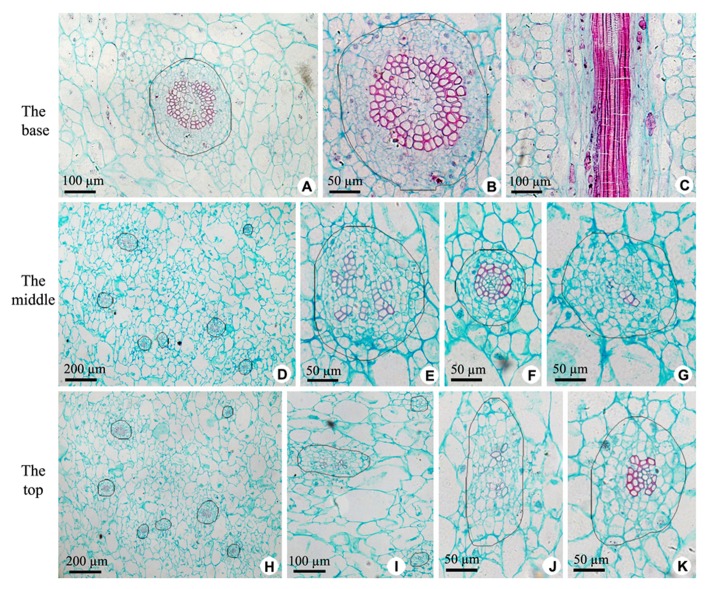
**Sections of central placenta at the base, in the middle and near the top of fruits, respectively. (A–C)** Views at the base. **(A)** Cross section of central placenta, showing one of vascular bundles. **(B)** Detailed view in **Figure 2A**, showing an amphicribral vascular bundle with the phloem surrounding the xylem. **(C)** Longitudinal section of central placenta, showing annular tracheids in xylem of amphicribral vascular bundle. **(D–G)** Views in the middle. **(D)** Cross section of central placenta, showing seven vascular bundles. **(E)** Detailed view of one of the amphicribral vascular bundles, showing the phloem surrounding the xylem, which is divided into several segments by parenchyma. **(F)** Detailed view of an amphicribral vascular bundle, showing the phloem surrounding the xylem and central parenchyma. **(G)** Detailed view of an amphicribral vascular bundle, showing the phloem surrounding the xylem of several tracheids. **(H–K)** Views near the top. **(H)** Cross section of central placenta, showing seven vascular bundles. **(I)** Three amphicribral vascular bundles with few tracheids, showing the phloem surrounding various amount of xylem. **(J)** Detailed view of left bundle in **Figure 2H**, showing branching bundle with branching xylem of few tracheids. **(K)** Detailed view of an amphicribral vascular bundle, showing the phloem surrounding the xylem.

The axile placenta of fruit is mainly formed of homogeneous, very large parenchymatous cells. Cross sections made through the placenta at the base (**Figures 2A–C**), in the middle (**Figures 2D–G**) and near the top (**Figures 2H–K**) of fruit indicate that vascular tissues are amphicribral bundles with secondary xylem in stellate-shaped arrangement, developed secondary phloem and obvious vascular cambium. After entering the axile placenta, these bundles immediately ramify and permeate the core (**Figures 2 and 5**), which include placental vascular bundles (PB) from the upper surface of receptacle and CP vascular bundles (CB) diverging from the former (**Figure 5**). With the extension of vascular bundles, xylem cross-sectional areas are getting smaller and smaller (**Figures 2B,E,J**), and the shapes of vascular cross section change from subcircular (**Figures 2A,B,E–G**) to suboblong (**Figures 2I–K**). Meanwhile, central parenchymatous areas of vascular bundles gradually decrease (**Figure 2**), but secondary phloem does not decrease in the proportion of vascular bundle, suggesting developed phloem provides an important structural basis for the axile transport of nutrients.

**FIGURE 3:**
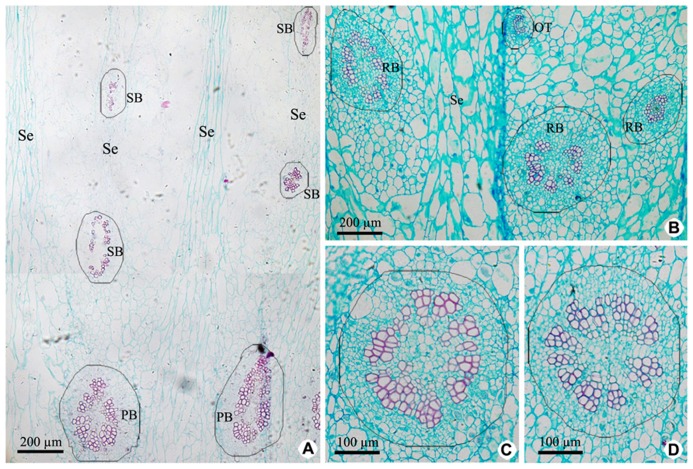
**Cross sections of peripheral placenta at the base of fruits showing the distribution, shape, type, and structure of amphicribral vascular bundles. (A)** Distribution of vascular bundles at placental periphery, showing placental vascular bundles at the periphery of axile placenta and septum vascular bundles in every other septum. **(B)** Cross section showing radial ovule-supplying branch and ovule trace embedded in parenchyma. **(C,D)** Detailed view of typical amphicribral placental vascular bundles with the phloem surrounding the xylem, which may be interrupted by parenchyma. Se, spetum; SB, septum vascular bundle; RB, radial ovule-supplying branch; OT, ovule trace.

Vascular tissues of PP include PB and its branches [radial ovule-supplying branch (RB), ovule trace (OT), and septum bundle (SB); **Figures 3A,B** and **5**], which are all amphicribral bundles, and their shapes, types, and structures are consistent with those in CP (**Figures 3** and **5**). PB, derived from the upper part of the receptacle, provide organic nutrients, water, and inorganic salts for ovules or seeds through RBs and OTs. SBs, distributed in every other septum, nourish septum tissue constituted by the two to six rows of cells arranged radially (**Figure 3A**). Vascular pattern in the middle and near the top (Figure not shown) is similar with that at the base of fruit (**Figure 3**).

**FIGURE 4:**
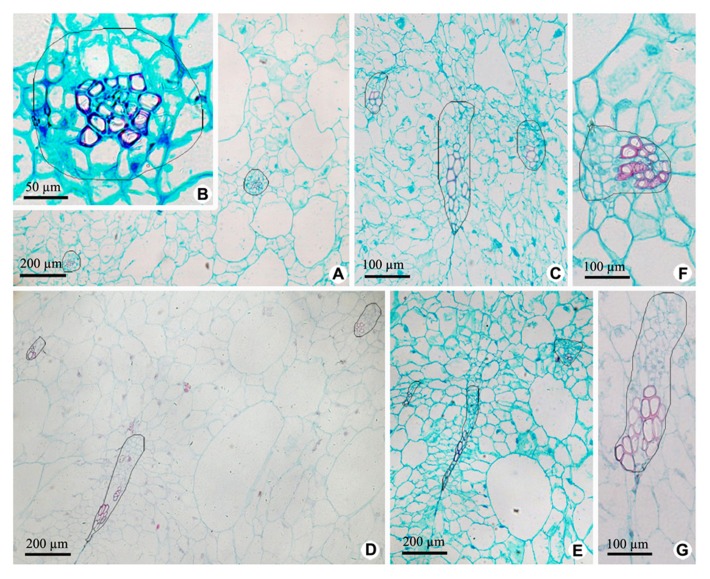
**Cross sections of outer pericarp at the base, in the middle and near the top of fruits. (A–C)** Views at the base. **(A)** Cross section of outer pericarp, showing two amphicribral bundles from lateral branch bundles. **(B)** Detailed view of the amphicribral bundle on the right side of **Figure 4A**. **(C)** Cross section of outer pericarp, showing three collateral bundles and their different shapes, which are all ovary wall vascular bundles. **(D,E)** Views in the middle. **(D)** Cross section of outer pericarp, showing three collateral bundles, in which two of them are lateral branch bundles (above), and the other is ovary wall vascular bundle (below). **(E)** Detailed view of different shapes of ovary wall vascular bundles, showing long and narrow bundle and nearly triangular view in cross section. **(F,G)**. Views near the top. **(F)** Detailed view of one lateral branch bundle, which is collateral. **(G)** Detailed view of an elongate ovary wall vascular bundle, which is collateral.

**FIGURE 5:**
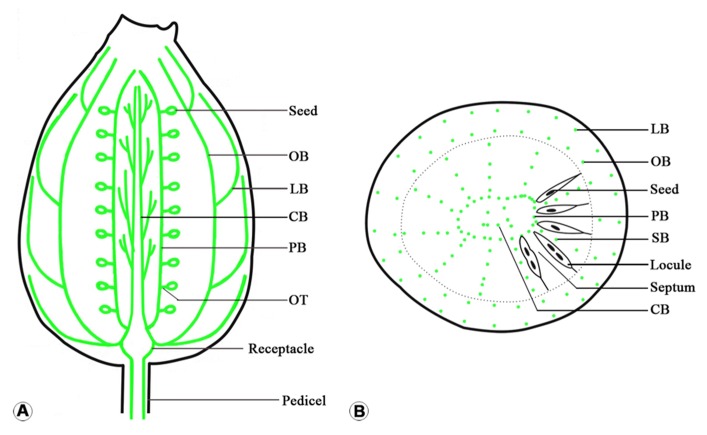
**Sketches of the vascular distribution of fruit of *Actinidia deliciosa *var *deliciosa *‘Qinmei.’ (A)** Longitudinal mid-section of fruit, showing the arrangement of the major vascular bundles in fruit. **(B)** Mid-cross section of fruit, showing the distributions of the major vascular bundles (green dots) in the fruit. Note septum vascular bundles occur in every other septum. CB, central placenta vascular bundle; LB, lateral branch bundle; PB, placental vascular bundle; OB, ovary wall vascular bundle; OT, ovule trace; SB, septum vascular bundle.

Vascular bundles in OP include ovary wall vascular bundle and its lateral branch bundle, like the midvein and secondary veins in a leaf. Ovary wall vascular bundles are from the receptacle (**Figures 4** and **5**). At the base of fruit, lateral branch bundles are amphicribral (**Figures 4A,B**), while ovary wall vascular bundles are collateral, which consist of less developed secondary xylem to the adaxial, vascular cambium with a weak activity and less developed secondary phloem to the abaxial (**Figure 4C**). In the middle and near the top of fruit, both of ovary wall vascular bundle and lateral branch bundle are collateral rather than amphicribral bundles (**Figures 4D–G**). As seen in **Figure 4**, cross-sectional areas of most ovary wall vascular bundles are bigger than those of lateral branch bundle, and the latter is oblong, while the former varies from long narrow shape to nearly triangular. In a bundle, cross-section area of the phloem is no less than that of the xylem (**Figures 4A–C**), highlighting the importance of the organic nutrient transport on fruit.

## DISCUSSION

Fruit vascular arrangement has important influence on fruit quality, and it has been investigated by previous authors. Examining the anatomical structure of kiwi fruit (*Actinidia chinensis* Planch. var. *chinensis*), Habart (1974) did not mention vascular bundles in CP and septa. Subsequently, based only on Habart’s work, Ferguson (1984) concluded that the central core and the septa should lack of vascular tissue, and this could further result in poor nutrient in these parts due to inadequate supplies of the immobile nutrient such as calcium. However, the present study shows that the central core and the every other septum have abundant secondary vascular tissue. In addition, in the determination of starch content in various parts of kiwi fruit and observation on their chloroplast ultrastructure, we have found starch contents continue to increase in axile placenta and inner pericarp, and are significantly higher in them than in OP at 140 days after anthesis (unpublished). These suggest that both of placental bundles and SBs have a strong function of transporting carbohydrates to meet the needs of these parts of fruit for nutrients. Further observation is necessary to testify whether there are anatomical differences between two cultivars of *Actinidia deliciosa *var. *deliciosa* ‘Qinmei’ and *Actinidia chinensis* Planch. var. *chinensis*.

It has been observed that kiwi fruit vasculature is composed of the multi-level branches arranged in order, and each part of fruit has corresponding bundles providing nutrients for it. PB and ovary wall vascular bundles enter the placenta and ovary wall from the receptacle through continuous extension and branching. Particularly, the ovary is superior, and all those to sepals, petals, and stamens from the base surface of receptacle are isolated from the placental branches, which all are amphicribral in organization. So, the placenta is a flower part independent of and separated from others, which are taken as foliar in nature. This would make the placenta a distinct organ.

According to the traditional botanical doctrines, the gynoecia of kiwifruit are constituted by multi-conduplicate carpel, and dorsal and ventral bundles in a carpel should have the same structure and type. However, our observations are not consistent with the theory. First, as shown in **Figures 2** **and 3**, all the vascular bundles are amphicribral in placenta and its periphery of kiwi fruit, implying an axial nature for the placental bundles, and most of them are collateral in ovary wall implying a leaf precursor for ovary wall. Second, as seen in **Figures 2A,B,E–G,K** and **3C,D**, stronger activity of vascular cambium in placental bundles, compared with that in ovary wall vascular bundles (**Figure 4**), further indicates that the placenta is an organ of the branch, and also implies ovary wall is a leaf organ, which usually lacks of secondary growth. Third, it can be inferred from traditional doctrine that, if septa are formed by the edges of carpel, and they all should have a uniform structure. Actually, our results show that SBs are distributed in every other septum (**Figure 3A**). This phenomenon is, however, understandable if the septum is taken as part of placenta, which, as a branch, includes vascular tissue and other tissue such as ground tissue. Fourth, combined with vascular patterns in CP and PP (**Figures 2D,H, 3A** and **5**), there is more than one ring of vascular bundles in placenta, which is very important. According to the traditional theory, carpel edge has only one ventral bundle involving in ovules bearing, and is impossible to form a few rings of vascular bundles, suggesting that the placenta is different from ovary wall in nature and origin. Fifth, in accordance with our current knowledge of botany, there is some of parenchyma in the center of xylem in amphicribral bundles with secondary growth, a typical configuration for eustele. So, amphicribral vascular with central parenchyma in placenta of kiwi fruit implies that the placenta is axial in nature (**Figures 2** and **3**), and is different from the ovary wall. This inconsistency between traditional doctrine and actual observations is not restricted to kiwi fruit. Amphicribral bundles have been shown to be related to ovules/placenta in various angiosperms [Papaveraceae (Kapoor, 1973,^1995^); Leguminosae in Figure 1 of Lersten and Don (1966); Winteraceae in Figure 2 of Tucker (1975); Solanaceae in Figure 1f of Dave et al. (1981); Gesneriaceae in Figures 11–13 of Wang and Pan (1998); Buxaceae in Figures 10Q and 82N of Von Balthazar and Endress (2002); Annonaceae in Figures 6G–K of Endress and Armstrong (2011)]. These families span from the magnoliids to terminal eudicot lineage in the tree of life for angiosperms (APG, 2009). However, little attention has been paid to this frequently seen phenomenon. The inconsistency between the traditional doctrine and observation in kiwi fruit and other plants casts serious doubt on the traditional doctrine.

The Unifying Theory states that the carpel in the classic sense is a composite organ comprising an ovule-bearing shoot (placenta) and a foliar part enclosing the shoot (Wang, 2010). This view is compatible with data from other fields of botany (Herr, 1995; Rounsley et al., 1995; Hickey and Taylor, 1996; Skinner et al., 2004; Dreni et al., 2007; Doyle, 2008; Zheng et al., 2010). If this theory is true, it can be presumed that there should be vascular bundles of radial symmetry (namely, amphicribral bundles) in the placenta. The amphicribral bundles in placenta of kiwi fruit in the present study provide important and crucial evidence favoring this theory. Moreover, studies on gene expression patterns in flowers of model plants including *Arabidopsis*, *Petunia*, and *Oryza* also indicate that STK, FBP7, FBP11, AGL11, and OsMADS13 are restricted to placenta/ovules (Angenent et al., 1995; Rounsley et al., 1995; Pinyopich et al., 2003; Dreni et al., 2007; Yoo et al., 2010; Li et al., 2011), while DL, CRC, and YABBY only to ovary wall (Yamaguchi et al., 2004; Dreni et al., 2007; Li et al., 2011). This implies that placenta is a distinct floral organ equivalent to a secondary shoot and independent of carpel and it is recruited onto ovary wall later in angiosperms (Angenent et al., 1995; Roe et al., 1997; Skinner et al., 2004). These conclusions are plausible considering that ovules are borne on fertile shoots in gymnosperms that have no carpels (Bierhorst, 1971; Biswas and Johri, 1997), and that ovule formation has nothing to do with carpels in mutant angiosperm (Lersten and Don, 1966; Dave et al., 1981; Skinner et al., 2004). Considering all, placenta in angiosperms is homologous with ovule-bearing branch, and the carpel wall with its subtending bract.

In the ovary wall vascular tissues, only lateral branch bundles are amphicribral at the base of fruit, implying an axis feature for the base of fruit wall, while all vascular bundles are collateral in other parts, implying a leaf characteristic for the middle and the top of fruit wall (**Figure 4**), that is to say, ovary wall of fruit, as leaf organ, has not completed the conversion process from axis to leaf at the base although well done in the middle and top portion. Studies on fossil plants provide some clues for the transformation from axis to leaf. *Psilophyton* and *Pauthecophyton* have distal axes with lateral, three-dimensional, synchronously dichotomous trusses. These lateral dichotomous trusses most probably performed a photosynthetic function similar to that performed by leaves, and distal divisions of the dichotomous trusses show a fundamentally leafy architecture, comprising a vascular bundle, parenchymatous mesophylls, and epidermis and lacking peripheral sterome (Banks et al., 1975; Gifford and Foster, 1989). Ultimate units of the branch-leaf complexes (BLC) of *Psilophyton dawsonii* and *Triloboxylon ashlandicum* show a uniform, fundamentally leafy histology, consisting of a vascular bundle (vein), parenchymatous mesophylls and epidermis (Banks et al., 1975; Scheckler, 1976). So, vascular anatomy of kiwi fruit wall shows a midway between axis and leaf, and thus sheds some new light on the derivation of leaves.

## Conflict of Interest Statement

The authors declare that the research was conducted in the absence of any commercial or financial relationships that could be construed as a potential conflict of interest.

## References

[B1] Angenent G. C. Franken J. Busscher M. van Dijken A. van Went J. L. Dons H. J. (1995). A novel class of MADS box genes is involved in ovule development in *Petunia*. *Plant Cell* 7 1569–1582 10.1105/tpc.7.10.1569 7580252 PMC161013

[B2] APG. (2009). An update of the Angiosperm Phylogeny Group classification for the orders and families of flowering plants: APG III. *Bot. J. Linn. Soc.* 161 105–121 10.1111/j.1095-8339.2009.00996.x

[B3] Banks H. P. Leclercq S. Hueber F. M. (1975). Anatomy and morphology of *Psilophyton dawsonii* sp.n. from the late Lower Devonian of Quebec (Gaspé), and Ontario, Canada. * Palaeontogr. Am.* 8 77–127

[B4] Bierhorst D. W. (1971). “Morphology of vascular plants,” in *The MacMillan Biology Series* eds Giles N. H. Torrey J. G. (New York: Macmillan Company)

[B5] Biswas C. Johri B. M. (1997). *The Gymnosperms*. Berlin:Springer-Verlag 494

[B6] Choat B. Gambetta G. A. Shackel K. A. Matthews M. A. (2009). Vascular function in grape berries across development and its relevance to apparent hydraulic isolation. *Plant Physiol.* 151 1677–1687 10.1104/pp.109.143172 19741048 PMC2773088

[B7] Clearwater M. J. Luo Z. Ong E. C. Blattmann P. Thorp T. G. (2011). Vascular functioning and the water balance of ripening kiwifruit (*Actinidia chinensis*) berries. *J. Exp. Bot.* 63 1835–1847 10.1093/jxb/err352 22155631 PMC3295381

[B8] Dave Y. S. Patel N. D. Rao K. S. (1981). Structural design of the developing fruit of *Nicotiana tabacum*. *Phyton* 21 63–71

[B9] Dichio B. Remorini D. Lang S. (2003). Developmental changes in xylem functionality in kiwifruit fruit: implications for fruit calcium accumulation. *Acta Hortic.* 610 191–195

[B10] Doyle J. A. (2008). Integrating molecular phylogenetic and paleobotanical evidence on origin of the flower. *Int. J. Plant Sci.* 169 816–843 10.1086/589887

[B11] Dreni L. Jacchia S. Fornara F. Fornari M. Ouwerkerk P. B. F. An G. (2007). The D-lineage MADS-box gene OsMADS13 controls ovule identity in rice. *Plant J.* 52 690–699 10.1111/j.1365-313X.2007.03272.x 17877710

[B12] Endress P. K. Armstrong J. E. (2011). Floral development and floral phyllotaxis in Anaxagorea (Annonaceae). *Ann. Bot.* 108 835–845 10.1093/aob/mBR201 21821626 PMC3177678

[B13] Ferguson A. R. (1984). Kiwifruit: a botanical review. *Hortic. Rev.* 6 1–64 10.1002/9781118060797.ch1

[B14] Gifford E. M. Foster A. S. (1989). *Morphology and Evolution of Vascular Plants*, 3rd Edn. New York: Freeman

[B15] Habart J. L. (1974). La baie de l’*Actinidia chinesis* Planch. var. *chinesis. Fruits* 29 191–207

[B16] Herr J. M. J. (1995). The origin of the ovule. *Am. J. Bot.* 82 547–564 10.2307/2445703

[B17] Hickey L. J. Taylor D. W. (1996). “Origin of angiosperm flower,” in *Flowering Plant Origin, Evolution and Phylogeny* eds Taylor D. W. Hickey L. J. (New York: Chapman and Hall) 176–231 10.1007/978-0-585-23095-5_8

[B18] Kapoor L. D. (1973). Constitution of amphicribral vascular bundles in capsule of *Papaver somniferum* Linn. *Bot. Gaz.* 134 161–165 10.1086/336698

[B19] Kapoor L. D. (1995). *Opium Poppy: Botany, Chemistry, and Pharmacology*. New York: Haworth Press Inc

[B20] Lersten N. R. Don K. W. (1966). The discontinuity plate, a definitive floral characteristic of the Psoraleae (Leguminosae). *Am. J. Bot.* 53 548–555 10.2307/2440004

[B21] Li H. Liang W. Yin C. Zhu L. Zhang D. (2011). Genetic interaction of OsMADS3, DROOPING LEAF, and OsMADS13 in specifying rice floral organ identities and meristem determinacy. *Plant Physiol.* 156 263–274 10.1104/pp.111.172080 21444646 PMC3091067

[B22] Marschner H. (1983). “General introduction to the mineral nutrition of plants,” in *Encyclopedia of Plant Physiology, *New Series Vol. 15A eds Läuchli A. Bielski R. L. (Berlin: Springer-Verlag) 5–60

[B23] Measham P. F. Gracie A. J. Wilson S. J. Bound S. A. (2010). Vascular flow of water induces side cracking in sweet cherry (*Prunus avium* L.). * Adv. Hortic. Sci. * 24 243–248 10.1400/153230

[B24] Morandi B. Rieger M. W. Corelli G. L. (2007). Vascular flows and transpiration affect peach (*Prunus persica* Batsch) fruit daily growth. *J. Exp. Bot.* 58 3941–3947 10.1093/jxb/erm248 18037679

[B25] Pinyopich A. Ditta G. Savidge B. Liljegren S. Baumann E. Wisman E. (2003). Assessing the redundancy of MADS-box genes during carpel and ovule development. *Nature* 34 85–88 10.1038/nature01741 12840762

[B26] Roe J. L. Nemhauser J. L. Zambryski P. C. (1997). TOUSLED participates in apical tissue formation during gynoecium development in *Arabidopsis*. *Plant Cell* 9 335–353 10.1105/tpc.9.3.335 9090879 PMC156922

[B27] Rounsley S. D. Ditta G. S. Yanofsky M. F. (1995). Diverse roles for MADS box genes in *Arabidopsis* development. *Plant Cell* 7 1259–1269 10.1105/tpc.7.8.1259 7549482 PMC160949

[B28] Scheckler S. E. (1976). Ontogeny of progymnosperms. I. shoots of Upper Devonian Aneurophytales. *Can. J. Bot.* 54 202–219 10.1139/b76-020

[B29] Schmid R. (1978). Reproductive anatomy of *Actinidia chinensis* (Actinidiaceae). *Bot. Jahrb. Syst. Pflanzengesch. Pflanzengeogr.* 100 149–195

[B30] Skinner D. J. Hill T. A. Gasser C. S. (2004). Regulation of ovule development. *Plant Cell * 16: S32-S45 10.1105/tpc.015933 15131247 PMC2643397

[B31] Tucker S. C. (1975). Carpellary vasculature and the ovular vascular supply in Drimys. *Am. J. Bot.* 62 191–197 10.2307/2441595

[B32] Von Balthazar M. Endress P. K. (2002). Reproductive structures and systematics of Buxaceae. *Bot. J. Linn. Soc.* 140 193–228 10.1046/j.1095-8339.2002.00107.x

[B33] Wang X. (2010). *The Dawn Angiosperms*. Heidelberg: Springer 10.1007/978-3-642-01161-0

[B34] Wang Y.-Z. Pan K.-Y. (1998). “Comparative floral anatomy of *Whytockia* (Gesneriaceae) endemic to China,” in *Floristic Characteristics and Diversity of East Asian Plants* eds Zhang A. L. Wu S. G. (Beijing: China Higher Education Press) 352–366

[B35] White P. J. (2001). The pathways of calcium movement to the xylem. *J. Exp. Bot.* 52 891–899 10.1093/jexbot/52.358.891 11432906

[B36] Yamaguchi T. Nagasawa N. Kawasaki S. Matsuoka M. Nagato Y. Hirano H. Y. (2004). The YABBY gene DROOPING LEAF regulates carpel specification and midrib development in *Oryza sativa*. *Plant Cell* 16 500–509 10.1105/tpc.018044 14729915 PMC341919

[B37] Yoo M.-J. Soltis P. S. Soltis D. E. (2010). Expression of floral MADS-box genes in two divergent water lilies: Nymphaeales and *Nelumbo*. *Int. J. Plant Sci.* 171 121–146 10.1086/648986

[B38] Zhang J. Liu Z.-M. Ma H.-P. Ma A.-P. (2009). Studies on anatomy and distribution of the vascular bundles in the peach fruits. *Acta Hotic. Sin.* 36 639–646 10.3724/SP.J.1035.2010.00639

[B39] Zheng H.-C. Ma S.-W. Chai T.-Y. (2010). The ovular development and perisperm formation of *Phytolacca* Americana (Phytolaccaceae) and their systematic significance in Caryophyllales. *J. Syst. Evol.* 48 318–325 10.1111/j.1759-6831.2010.00082.x

